# Virus and Virus-like Pathogens in the Grapevine Virus Collection of Croatian Autochthonous Grapevine Cultivars

**DOI:** 10.3390/plants11111485

**Published:** 2022-05-31

**Authors:** Darko Vončina, Alfredo Diaz-Lara, Darko Preiner, Maher Al Rwahnih, Kristian Stevens, Snježana Jurić, Nenad Malenica, Silvio Šimon, Baozhong Meng, Edi Maletić, Hrvoje Fulgosi, Bogdan Cvjetković

**Affiliations:** 1 Department of Plant Pathology, Faculty of Agriculture, University of Zagreb, 10000 Zagreb, Croatia; 2 Centre of Excellence for Biodiversity and Molecular Plant Breeding (CroP-BioDiv), 10000 Zagreb, Croatia; dpreiner@agr.hr (D.P.); emaletic@agr.hr (E.M.); 3 School of Engineering and Sciences, Tecnologico de Monterrey, Campus Queretaro, Queretaro 76130, Mexico; diazlara@tec.mx; 4 Department of Viticulture and Enology, Faculty of Agriculture, University of Zagreb, 10000 Zagreb, Croatia; 5 Foundation Plant Services, University of California-Davis, Davis, CA 95616, USA; malrwahnih@ucdavis.edu (M.A.R.); kastevens@ucdavis.edu (K.S.); 6 Departments of Computer Science and Evolution and Ecology, University of California-Davis, Davis, CA 95616, USA; 7 Division of Molecular Biology, Ruđer Bošković Institute, 10000 Zagreb, Croatia; snjezana.juric1980@gmail.com (S.J.); Hrvoje.Fulgosi@irb.hr (H.F.); 8 Division of Molecular Biology, Faculty of Science, University of Zagreb, 10000 Zagreb, Croatia; nenad.malenica@biol.pmf.hr; 9 Directorate for the Professional Support for the Development of Agriculture, Ministry of Agriculture, 10000 Zagreb, Croatia; silvio.simon@mps.hr; 10 Department of Molecular and Cellular Biology, University of Guelph, Guelph, ON N1G 2W1, Canada; bmeng@uoguelph.ca; 11 Department of Agricultural Zoology, Faculty of Agriculture, University of Zagreb, 10000 Zagreb, Croatia; bcvjetkovic@agr.hr

**Keywords:** grapevine virus, viroid, “flavescence dorée”, ELISA, Western blot, RT-PCR, cloning, sequencing, LAMP

## Abstract

Grapevine collections play an important role, especially in the study of viruses and virus-like pathogens. In 2009, after an initial ELISA screening for eight viruses (arabis mosaic virus, grapevine fanleaf virus, grapevine fleck virus, grapevine leafroll-associated viruses 1, 2, and 3, and grapevine viruses A and B), a collection of 368 grapevine accessions representing 14 different Croatian autochthonous cultivars and containing single or mixed infection of viruses was established to further characterize the viral pathogens. Subsequently, Western blot, RT-PCR, cloning, and sequencing revealed that grapevine rupestris stem pitting-associated virus was frequently found in accessions of the collection, with isolates showing substantial genetic diversity in the helicase and coat protein regions. High-throughput sequencing of 22 grapevine accessions provides additional insight into the viruses and viroids present in the collection and confirms the fact that Croatian autochthonous grapevine cultivars have high infection rates and high virome diversity. The recent spread of “flavescence dorée” phytoplasma in Europe has not spared the collection. After the first symptoms observed in 2020 and 2021, the presence of phytoplasma was confirmed by LAMP in six grapevine accessions and some of them were lost. Single or multiple viruses and viroids, as well as own rooted grapevines in the collection, make the plants susceptible to various abiotic factors, which, together with the recent occurrence of “flavescence dorée”, makes the maintenance of the collection a challenge. Future efforts will be directed towards renewing the collection, as 56% of the original collection has been lost in the last 13 years.

## 1. Introduction

Collections of plant viruses and virus-like agents, in vivo in their host plants, are a crucial prerequisite for the study of multiple aspects of the pathogen, especially for the development of robust detection methods and for epidemiological and ecological studies. Plant collections occupy a special position when dealing with pathogens that cannot be cultured on artificial medium, such as viruses and viroids. Since grapevine (*Vitis vinifera* L.) is one of the most widespread woody crops with significant economic importance, much work has been carried out on various pathogens, including viral agents. Eighty-six viruses have been confirmed to infect grapevine by 2020, with varying adverse effects ranging from unknown to very important and associated with substantial economic losses [1]. Globalization, the demands of grapevine growers and stakeholders, rapid advances in numerous areas of research, and the specific biology of pathogens, among other factors, have led to the establishment of many grapevine virus collections around the world. One of the oldest is the UC-Davis grapevine virus and virus-like disease collection (USA) established in the 1960s [2]. Other collections include the Agroscope grapevine virus collection at Nyon (Switzerland) and the collection of the ARC-Plant Protection Research Institute, Pretoria (South Africa). Due to various requests, especially from grapevine and nursery producers, but also for educational and scientific purposes, a grapevine virus collection was established in 2009 at the University of Zagreb Faculty of Agriculture. During this period, the collection counted 368 grapevine accessions of Croatian autochthonous grapevine varieties collected from different viticultural regions in the coastal area within the framework of several clonal selection programs. In the period from 2009 to 2022, the collection was used for identification and characterization of different viruses such as grapevine leafroll-associated virus 2 (GLRaV-2), GLRaV-4 like group, grapevine viruses B, D, E, and F (GVB, D, E, F), grapevine redglobe virus (GRGV), grapevine Syrah virus 1 (GSyV-1), and grapevine rupestris vein feathering virus (GRVFV), which were detected for the first time in Croatia [3,4,5]. Recently, using high-throughput sequencing (HTS) technology, GVG, GVL, and grapevine badnavirus 1 (GBV-1) were reported from the accessions present in the collection [6,7], showing the great virome diversity present in these vines.

This article describes the introduction of new methods and techniques for the detection of grapevine viruses and virus-like pathogens and reviews the additional studies that have been conducted on plants from the Croatian collection but have not been published. The implications and uses of these types of germplasm collections are also discussed. Finally, special emphasis is placed on recent challenges related to the maintenance of this collection.

## 2. Results

### 2.1. Virus Screening by ELISA and Collection Establishment

**Figure 1:**
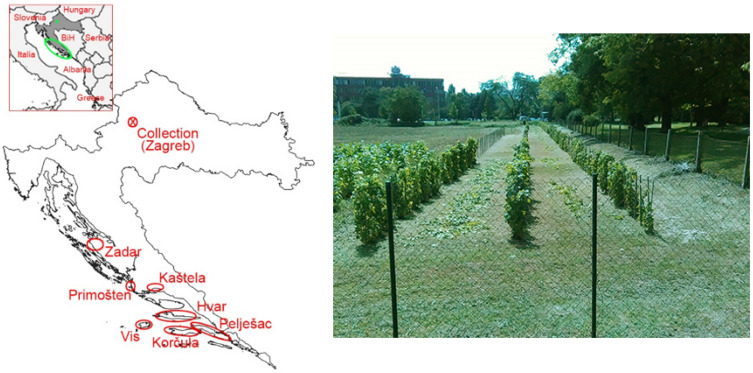
Location of the Croatian grapevine virus collection and the main growing areas from which cuttings were collected (**left**) and later planted in the collection (**right**).

As a result of ELISA virus screening targeting eight different viruses (arabis mosaic virus—ArMV, grapevine fanleaf virus—GFLV, grapevine fleck virus—GFkV, grapevine virus A—GVA, GVB, GLRaV-1, 2, and 3), 368 grapevine accessions were selected based on virus significance, infection status (single or mixed), site of origin, and cultivar; later, their rooted cuttings were included in the grapevine virus collection (Figure 1).

Most of the grapevines (35%) were from the Kaštela region, as this region and its autochthonous cultivars were found to be highly infected with various combinations of viruses. Overall, ArMV, GFLV, GFkV, GLRaV-1, 2, 3, GVA, and GVB were present in 12 (3.3%), 61 (16.6%), 87 (23.6%), 131 (35.6%), 29 (7.9%), 312 (84.8%), 203 (55.2%), and 22 (5.9%) grapevine accessions, respectively, which roughly reflects the sanitary situation found in the original sites/vineyards from which the cuttings were collected. According to the number of viruses in each accession, eight (2.2%) plants were free of viruses tested, 85 (23.1%) had a single virus infection, 114 (30.9%) were infected with two viruses, 109 (29.6%) with three, 42 (11.4%) with four, eight (2.2%) with five, while one (0.3%) grapevine accession was infected with six viruses. A detailed overview of all grapevine accessions included in the collection and their sanitary status as determined by ELISA can be found in Supplementary Table S1.

### 2.2. Grapevine Rupestris Stem Pitting-Associated Virus (GRSPaV) Screening by Western Blot and Other Molecular Methods, including Phylogenetic Analysis

As part of the information gap regarding GRSPaV infection, one of the most widespread grapevine viruses, screening of 26 accessions from the collection was performed by Western blot, RT-PCR, cloning, and Sanger sequencing. For this purpose, plants from different locations in the coastal region were selected, and a comparison of detection with two primary antibodies (As7-276 and As2003) in the Western blot and two primer sets (RSP13/RSP14 targeting the helicase gene and 48V/49C targeting the coat protein gene) for detection by RT-PCR was performed. Western blot with antibodies As7-276 developed a clear signal in 20 samples, with two other samples showing a weak signal (Babic 113, Vlaska 137), while As2003 clearly detected GRSPaV in 21 samples, with another sample showing a weak signal (Vlaska 137). In the same three samples (Marastina 034, Plavac mali 128, and Dobricic 101), the virus was not detected by either antibody, while Babic 113 gave a weak signal with As7-276 and no signal with As2003. Including the weak positives, the detection rate for As7-276 and As2003 was 23/26 (88.5%) and 22/26 (84.6%), respectively.

The primer pair RSP13/RSP14 used in RT-PCR confirmed the presence of the virus in all accessions tested, while the primer pair 48V/49C failed to detect the virus in Plavac mali 234, giving a diagnostic sensitivity of 96.2%. A detailed overview of the detection efficiencies using different antibodies in Western blot and different primer sets in RT-PCR can be found in Table 1 and Supplementary Figure S1.

Cloning and Sanger sequencing of the 299 nts (99 aa) long segment of the helicase gene and the 289 nts (96 aa) long segment of the coat protein gene was performed. Excluding the primer sequences, sequence analysis of the helicase segment revealed pairwise identity in the range of 87.62–98.99% and 94.94–100% at the nt and aa levels, respectively. The same analysis of the coat protein segment revealed pairwise identity in the range of 94.11–100% and 95.83–100% at the nt and aa levels, respectively (Table 2).

Comparison of Croatian GRSPaV isolates with sequences available in GenBank showed high similarity with other isolates from other parts of the world, especially with those from South Africa, in both the replicase and the coat protein regions. A detailed overview of GRSPaV isolates from other countries most similar to those found in Croatia can be found in Table 3.

**Figure 2:**
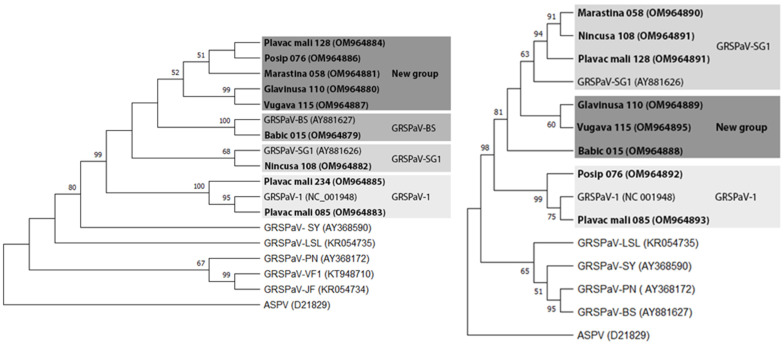
Phylogenetic trees of the Croatian (showed in bold) and foreign GRSPaV sequence variants, with corresponding GenBank accession numbers, based on the nucleotide sequence of the part of the helicase (**left**) and the part of the coat protein region—CP (**right**). In addition to the Croatian isolates, those representing type members of different groups were used, as well as the apple stem pitting virus isolate PA66 (D21829) as rooting outgroup. Phylogenetic analyses were performed using maximum likelihood method (ML). Bootstrap values of 50% or more (out of 1000 replications) are indicated on the branches.

Phylogenetic analysis showed the Tamura-3 parameter model with invariant sites (I) and Kimura-2 parameter with gamma distribution as the best models for nucleotide substitution for the partial helicase and the partial CP region, respectively. The phylogenetic tree of helicase poisoned isolate Babic 015 within the GRSPaV-BS group, isolate Nincusa 108 within the GRSPaV-SG1 group, and Plavac mali 085 and 234 within the GRSPaV-1 group, whereas the other isolates formed a separate group. According to the CP phylogenetic tree, isolates Marastina 058, Nincusa 108, and Plavac mali 128 clustered within the GRSPaV-SG1 group, Posip 076 and Plavac mali 085 clustered within the GRSPaV-1 group, while three other isolates (Glavinusa 110, Vugava 115, and Babic 015) clustered separately (Figure 2).

### 2.3. High-Throughput Sequencing (HTS)

To gain better insight into the composition of viruses and viroids in grapevines from the collection, 22 accessions were selected for screening by HTS on the Illumina NextSeq 500 platform. For selected grapevines, results from HTS confirmed ELISA results obtained ca 10 years ago and detected the presence of 10 additional viruses not included in the original screening: GVE, GVF, GVG, GVT, GLRaV-4 (including strains 5, 6, 9), GRSPaV, GDefV, GRLDaV, GRVFV, and GSyV-1. On the other hand, there were differences in the presence of some viruses that were not determined by ELISA, but were detected by HTS, in particular GLRaV-1 (Plavac mali 084, Plavac mali 275), GVA (Dobricic 021, Plavac mali 275, Plavac mali 084, Posip 056), GFLV (Vugava 036, Plavac mali 235), and GFkV (Vlaska 150, Babic 055, Marastina 016, Babica 122, Pošip 123, and Posip 084). HTS analysis confirmed the high incidence of GRSPaV infection in the collection (81.8% of samples screened by HTS), which was already detected in other grapevines by Western blot and RT-PCR. In addition, the following viroids were identified in HTS-screened samples: hop stunt viroid (HSVd) and grapevine yellow speckle viroids 1 and 2 (GYSVd-1 and GYSV-2) (Table 4, Supplementary Table S2).

### 2.4. Loop-Mediated Isothermal Amplification (LAMP) Screening for “Flavescence Dorée”

After the first appearance of “flavescence dorée” (FD)-like symptoms in the collection during the 2020 growing season, followed by rapid spread, symptomatic plants were tested for the presence of FD in September 2020 and 2021 by LAMP. The presence of FD was confirmed in six (40%) of the fifteen samples tested. Two grapevine accessions were tested in two consecutive growing seasons, one resulting as negative (Vlaska 143) and the other positive (Plavac mali 313) in both seasons. Grapevine accessions Grk 003 and Mladenka 148 showed FD-like symptoms in 2020, but did not show symptoms in 2021, while Plavac mali 272, Vlaska 131, and 150 were lost during the winter 2020/21 (Table 5).

## 3. Discussion

Following the practice of other countries, where viticulture is an important economic sector, a collection of grapevine viruses was established in 2009 at the experimental field of University of Zagreb Faculty of Agriculture. Initially, the collection served as a source of positive and negative controls in diagnostic tests for economically important viruses, but over the time it has also been used for other experiments and investigations, such as studies on genetic diversity, vector transmission, and alternative hosts. Consequently, this grapevine collection has allowed cooperation between different scientific institutions (national and international) in the form of material exchange that can greatly improve current knowledge and diversity of grapevine pathogens.

After the establishment of the Croatian grapevine virus collection and ELISA screening for eight viruses, further studies showed a high infection rate with GRSPaV, a virus with known global distribution [8]. The primer set RSP13/RSP14, targeting the helicase region, detected the virus in all 26 grapevine accessions, confirming its ability to detect different virus variants [9,10,11,12], while RT-PCR, using the primer set 48V/49C targeting the coat protein, was a bit less effective and failed to detect the virus only in grapevine accession Plavac mali 234. According to the results of this study, serological methods based on Western blot were, as expected, less efficient than RT-PCR. The polyclonal antiserum As2003 showed a clearer result compared to As7-276. The lower detection efficiency of the Western blot (88.5% with As7-276 and 84.6% with As2003), compared to RT-PCR, may be the consequence of the variability of the virus titer during the season, grapevine cultivar, or the type of the plant tissue used [13,14].

The Sanger sequencing results revealed the substantial genetic diversity of the Croatian GRSPaV. Interestingly, the isolates from cv. Plavac mali (85, 128, and 234) were found to be identical in the helicase region, and 085 and 128 were found to be identical in the partial coat protein, although each came from a different region (Korčula Island, Pelješac Peninsula, and Hvar Island, respectively). Compared with the other sequences in GenBank, the Croatian GRSPaV isolates showed the greatest similarity in the helicase region with isolates from South Africa, Italy, Canada, Slovakia, and France. Interestingly, in the case of Plavac mali 128, the most similar isolate was from Italy, derived from the cultivar Pagadebit, which is considered synonymous with Plavac mali. In the coat protein region, Croatian GRSPaV isolates had slightly lower diversity, comparable to isolates from South Africa, Tunisia, Brazil, China, Italy, and France. The high genetic diversity of some isolates was also confirmed by phylogenetic analyses of the part of the helicase gene, which placed five Croatian isolates (Plavac mali 128, Posip 076, Marastina 058, Glavinusa 110, Vugava 115) in a separate group, while three isolates (Glavinusa 110, Vugava 115, and Babic 015) formed a separate group according to the partial coat protein region. This is consistent with the results of other studies confirming the high diversity also found in isolates of this virus from different parts of the world [9,11,12].

The introduction of HTS in grapevine virus diagnostics as of 2009 [15] represents a significant improvement in all aspects of grapevine virology, especially in the identification of novel viruses and better insight into the complete sanitary status of the tested plants. The HTS analysis performed on 22 grapevine accessions in 2019 proved superior to ELISA results performed on eight viruses (ArMV, GFLV, GFkV, GLRaV-1, 2, 3, GVA, and GVB) in 2007–2008. It detected all viruses confirmed by ELISA, except ArMV in the grapevine accession Marastina 037, and provided additional information on health status. Thus, HTS detected GFLV in two grapevine accessions not detected by ELISA (Plavac mali 235 and Vugava 036), GFkV in six accession (Babica 122, Babic 055, Marastina 016, Posip 084, Posip 123, and Vlaska 150), GLRaV-1 in two additional accessions (Plavac mali 084 and 275), and GVA in four other accessions (Dobricic 021, Plavac mali 084, 275, and Posip 056). As previously mentioned, the only difference in favor of ELISA was the detection of ArMV in Marastina 037, which was not determined by HTS. Since ArMV is serologically related to GFLV, which is also present in the mentioned grapevine accession, it is possible that the ELISA antibodies reacted with GFLV, resulting in a false positive result for ArMV. In the interpretation of the ELISA and HTS results and their differences, it must also be taken into account the 11-year period between the application of these two methods, the period during subsequent infections through vectors, particularly mealybugs, whose presence was observed in the plantation, and which could transmit viruses of the leafroll complex and GVA. Moreover, since soil analysis on nematodes was never performed, the possibility of GFLV transmission via the nematode *Xiphinema index* cannot be excluded, especially in the plants Babica 122, Marastina 016, Posip 123, and Vugava 036, since their neighboring plants were infected with GFLV (Supplementary Table S1). The high abundance of GRSPaV was also confirmed by HTS in 18 of 22 tested grapevines (81.8%). Finally, the HTS analyses indicated the presence of 10 other viruses (GVE, F, G, T, GLRaV-4 group, GDefV, GRLDaV, GBV-1, GRVFV, GsyV-1) and three viroids (GYSV-1, GYSV-2, and HSVd). These results are consistent with other previously conducted studies demonstrating high viral diversity in the autochthonous grapevines [5,6,16].

Recently, the occurrence of the FD phytoplasma has become a major challenge in the vineyard management. In the last 30 years, FD has become a dangerous problem in Europe, and large-scale spread has been reported from several countries, along with the presence of its vector, the leafhopper *Scaphoideus titanus* Ball [17]. The presence of FD was confirmed in 4 out of 7 symptomatic grapevines examined in 2020 and in 3 out of 10 grapevine accessions in 2021. Although infected Plavac mali 313 survived two seasons, three FD-positive accessions were lost in 2020 (Plavac mali 272, Vlaska 131, and 150) due to FD infection. FD-like symptoms observed in other plants that turned to be negative, according to LAMP, could be due to infections with other representatives of grapevine yellows associated with similar symptoms, such as “*Candidatus* Phytoplasma solani”. In the future, the main focus will be on the further spread of symptoms associated with grapevine yellows and the removal of infected plants as a prophylactic measure.

As shown in this study, in vivo collections play a unique role in various aspects of virus and virus-like pathogen research. The 13-year experience has shown that the maintenance of such collections is even more challenging, as infection with viruses, viroids, and phytoplasmas negatively affects the tolerance of plants to abiotic factors such as low temperatures, especially late frosts in spring, high temperatures in summer, often accompanied by water shortages, and biotic factors such as fungal or pseudofungal oomycete infection (powdery mildew, downy mildew) and, as the grapevine grows on its own roots, phylloxera. Evidence of the very demanding care is that in the 13 years after establishment, 207 out of 368 (56%) grapevine accessions were lost for various reasons (Supplementary Table S1). In order to save the collection from further deterioration, 150–200 new accessions of autochthonous cultivars currently used for research on emerging viruses, mainly grapevine virus G and grapevine badnavirus 1, will be added to the virus collection to provide a prerequisite for further research.

## 4. Materials and Methods

### 4.1. ELISA Screening and Plant Material Used for the Grapevine Virus Collection

In 2007 and 2008, several extensive studies focused on clonal and sanitary selection of autochthonous grape varieties were conducted in the Croatian coastal viticultural region. In these field surveys grapevines were tested for eight different viruses (ArMV, GFLV, GLRaV-1, 2, and 3, GVA, GVB, and GFkV) using ELISA kits from Agritest (Valenzano, Italy). The sample source was 0.1 g of phloem scrapings taken from well-wooded cuttings collected during dormancy, which were pulverized in a sterile mortar with a pestle and the addition of liquid nitrogen. The pulverized tissue thus obtained was diluted with extraction buffer and all other ELISA steps were performed according to the manufacturer’s instructions. The OD values for each sample were determined using the EL 800 spectrophotometer (BioTek, Winooski, VT, USA) at a wavelength of 405 nm two hours after addition of the substrate p-nitrophenyl phosphate (Sigma-Aldrich, Saint Louis, MO, USA). Samples/grapevines with an OD value that were at least two times the average value of the negative controls were considered positive. Based on the ELISA results, 368 grapevines were selected according to their sanitary status (no virus, single or mixed infections), place of origin, and cultivar, and their cuttings were placed in plastic containers filled with planting medium/peat to be rooted under the greenhouse conditions. A year later, the potted accessions were moved to an open area, and in 2009, the grapevines were planted at the current site, part of the experimental field located in the vicinity of the campus of University of Zagreb Faculty of Agriculture.

### 4.2. Grapevine Rupestris Stem Pitting-Associated Virus (GRSPaV) Screening by Western Blot and Other Molecular Methods, including Phylogenetic Analysis

To cover a broader region and potential wider genetic variability, this part of the study was conducted on a total of 26 grapevines/plants from 26 different sites/vineyards comprising 12 autochthonous cultivars (Babica, Babic, Dobricic, Glavinusa, Ljutun, Marastina, Mladenka, Nincusa, Plavac mali, Posip, Vlaska, and Vugava) from the collection. Mentioned grapevines were subjected to grapevine rupestris stem pitting-associated virus (GRSPaV) analysis by Western blot and RT-PCR followed by cloning and sequencing. In both tests (Western blot and RT-PCR), lyophilized tissue of cv. Refošk infected with GRSPaV (courtesy of Urška Čepin, National Institute of Biology, Ljubljana, Slovenija) was used as positive control, while tissue from cv. Babic free from mentioned virus was used as a negative control.

Western blot was performed according to the standard protocol: proteins from 100 mg of young leaf petioles collected during the beginning of vegetation (March) were isolated according to the previously described method [14]. Briefly, proteins were resolved by SDS-PAGE and transferred to a nitrocellulose membrane using the submerged electroblotting transfer technique. Membranes were incubated with a 1:1000 dilution of the primary antibody (As7-276 or As2003) in blocking buffer at 4 °C overnight. Goat anti-rabbit IgG conjugated to horseradish peroxidase (HRP) at 1:5000 dilution was used as the secondary antibody. Protein signals were detected using the enhanced chemiluminescence assay.

In the case of RT-PCR, total RNA was extracted from 0.1 g of cortical scrapings taken from well-wooded canes using the RNeasy Plant Mini Kit (Qiagen, Hilden, Germany). The RNA was used for one-step RT-PCR detection with two sets of primers: RSP13/RSP14, which amplify 339 bp fragment of the helicase domain [18], and 48V/49C, specific for an internal part of the coat protein gene resulting in 331 bp product [19]. Two μL of total RNA was used as a template in RT-PCR which was performed using a OneStep RT-PCR kit (Qiagen, Hilden, Germany) according to manufacturer’s instructions in a 25 μL RT-PCR-mixture using 0.6 μM of each primer and cycling conditions: reverse transcription at 50 °C for 30 min followed by the initial PCR activation step at 95 °C for 15 min and 40 cycles of multiplication (94 °C for 45 s, 55 °C for 1 min, 72 °C for 1 min) and final extension at 72 °C for 10 min. Reaction products were analyzed by electrophoresis in 1.5% agarose gel stained in ethidium bromide (0.75 mg/mL) and visualized on Gel Doc XR (Bio-Rad Laboratories, Hercules, CA, USA) using Quantity One program package (Bio-Rad Laboratories, Hercules, CA, USA). RT-PCR amplicons obtained from nine (RSP13/14) and eight (48V/49C) grapevines were purified with GenElute PCR Clean-Up kit (Sigma-Aldrich, Saint Louis, MO, USA) according to manufacturer’s instructions and cloned into the pGEM-T Easy cloning vector (Promega, Madison, WI, USA) according to manufacturer’s instructions with minor modifications. Thus, 10 ng of each PCR amplicon was used in a ligation reaction performed at 22 °C for 1 h, followed by a 4 °C overnight incubation. Three microliters of ligation mixtures were transformed into homemade XL1-Blue electro-competent cells. Upon the blue/white selection, plasmid DNA was isolated and digested with restriction endonucleases EcoRI (MBI Fermentas, Vilnius, Lithuania) or BstXI (New England Biolabs, Beverly, MA, USA) to confirm the insertion. Cloned products were sequenced as previously described [4] on an ABI 3130 Genetic Analyzer (Applied Biosystems, Foster City, CA, USA) in both directions. Sequences were edited using BioEdit Sequence Alignment Editor ver. 7.0.9.0 [20] and after primers removal, sequences of 299 (RSP13/RSP14) and 289 (48V/49C) nts were aligned with each other and compared to the other isolates from the GenBank using the ClustalW program [21]. Therefore, the sequences of the Croatian GRSPaV isolates were used to construct phylogenetic trees for both regions (helicase and CP) using different phylogroups, as suggested by Meng and Rowhani [8] for the helicase region and Meng and Rowhani [8] and Terlizzi et al. [22] for the CP region. The best model of nucleotide substitution and the construction of phylogenetic trees were performed using the maximum likelihood method (ML) with 1000 bootstrap replicates in MEGA11 software [23]. The following isolates were used to construct phylogenetic trees: GRSPaV-1 (NC_001948), GRSPaV- SY (AY368590), GRSPaV-BS (AY881627), GRSPaV-JF (KR054734), GRSPaV-LSL (KR054735), GRSPaV-VF1 (KT948710), GRSPaV-PN (AY368172), GRSPaV-SG1 (AY881626), and the apple stem pitting virus isolate PA66 (D21829) as the rooting outgroup.

### 4.3. High-Throughput Sequencing (HTS)

Total nucleic acid (TNA) extracts were prepared from 22 grapevine accessions as previously described [24]. TNA was isolated from petioles of selected grapevines. Aliquots of TNA from source samples were subjected to ribosomal RNA (rRNA) depletion and complementary DNA (cDNA) library construction employing a TruSeq Stranded Total RNA with Ribo-Zero Plant kit (Illumina, San Diego, CA, USA). Later, cDNA libraries were sequenced using the Illumina NextSeq 500 platform located at the UC-Davis Genome Center. Sequencing reads were demultiplexed and adapter trimmed via bcl2fastq Conversion Software (Illumina). Trimmed reads were de novo assembled into contigs using SPAdes [25]. Generated contigs were compared against the complete non-redundant GenBank virus database using BLASTn for nts and BLASTx for aa, providing the annotation used for viral agent identification. Contigs above 500 nts, and with identity over the 80% at the nt or aa level were taken as threshold in order to call the virus species.

### 4.4. Loop-Mediated Isothermal Amplification (LAMP) Screening for “Flavescence Dorée”

Screening for the presence of FD phytoplasma was completed in 2020 and 2021. Samples were selected according to the symptoms typical for grapevine yellows: yellowing/reddening discoloration and downward rolling of laminae. Extraction of DNA was performed from leaf petioles and main veins of symptomatic leaves using the Plant Material Lysis Kit (OptiGene, West Sussex, UK) from seven grapevine accessions in 2020 and from 10 accessions in 2021. Detection of FD was performed as previously described [26] using commercial FD assay mix and plant control assay (OptiGene, Horsham, UK) with primers targeting cytochrome oxidase as internal control, both used according to manufacturer’s instructions in 25 µL of reaction mixture: 15 µL of isothermal master mix, 5 µL of primer mix, and 5 µL of template DNA. LAMP reactions were performed on Genie III (OptiGene, Horsham, UK) using the following conditions: an isothermal amplification step of 65 °C for 30 min followed by an anneal step from 98 °C to 80 °C at 0.05 °C/s. Each test included a positive (FD-infected grapevine) and a negative (FD-free grapevine) control. Samples with an exponential or “S” amplification curve and melting temperatures around 85.0 °C (±0.3 °C) were considered positive.

## Figures and Tables

**Table 1:** Detection of grapevine rupestris stem pitting-associated virus (GRSPaV) by Western blot (antibodies As7-276 and As2003) and RT-PCR (primers RSP13/RSP14 and 48V/49C) together with samples whose RT-PCR products were cloned and sequenced in both directions (N/A—not applicable because RT-PCR products were not sequenced).

No. on the Gels	Cultivar/Grapevine Accession	Western Blot		RT-PCR		Cloning/ Sequencing
		As7-276	As2003	RSP13/RSP14	48V/49C	
1.	Babic 015	+	+	+	+	+
2.	Babic 056	+	+	+	+	N/A
3.	Babic 113	+/−	−	+	+	N/A
4.	Babic 076	+	+	+	+	N/A
5.	Marastina 034	−	−	+	+	N/A
6.	Marastina 058	+	+	+	+	+
7.	Marastina 101	+	+	+	+	N/A
8.	Posip 076	+	+	+	+	+
9.	Vugava 060	+	+	+	+	N/A
10.	Vugava 115	+	+	+	+	+
11.	Plavac mali 013	+	+	+	+	N/A
12.	Plavac mali 085	+	+	+	+	+
13.	Plavac mali 234	+	+	+	−	+
14.	Plavac mali 286	+	+	+	+	N/A
15.	Plavac mali 111	+	+	+	+	N/A
16.	Plavac mali 128	−	−	+	+	+
17.	Plavac mali 159	+	+	+	+	N/A
18.	Plavac mali 181	+	+	+	+	N/A
19.	Plavac mali 219	+	+	+	+	N/A
20.	Babica 106	+	+	+	+	N/A
21.	Dobricic 101	−	−	+	+	N/A
22.	Glavinusa 110	+	+	+	+	+
23.	Ljutun 167	+	+	+	+	N/A
24.	Mladenka 154	+	+	+	+	N/A
25.	Nincusa 108	+	+	+	+	+
26.	Vlaska 137	+/−	+/−	+	+	N/A

**Table 2:** Comparison of nucleotide and amino acid identities of GRSPaV isolates from nine different grapevine accessions. Sequences were obtained by cloning and include a 299 nts/99 aa long portion of the helicase and a 289 nts/96 aa long portion of the coat protein after primer removal, obtained by RT-PCR using RSP13/RSP14 and 48V/49C primer sets, respectively. Values below the diagonal correspond to nucleotide identity (blue shaded), whereas values above the diagonal correspond to amino acid identity (green shaded). Identity ranges are shown by different shades of blue/green (categories 85–89.9%, 90–94.9%, 95–99.9%, 100%).

Helicase (RSP13/RSP14)									
Babic 015	100%	96.96%	97.97%	95.95%	97.97%	97.97%	97.97%	98.98%	97.97%
Glavinusa 108	92.64%	100%	96.96%	94.94%	98.98%	98.98%	98.98%	97.97%	98.98%
Marastina 058	92.64%	96.65%	100%	95.95%	97.97%	97.97%	97.97%	98.98%	97.97%
Nincusa 108	88.29%	90.63%	92.64%	100%	95.95%	95.95%	95.95%	96.96%	95.95%
Plavac mali 085	88.29%	90.63%	92.3%	87.62%	100%	100%	100%	98.98%	97.97%
Plavac mali 128	88.29%	90.63%	92.3%	87.62%	100%	100%	100%	98.98%	97.97%
Plavac mali 234	88.29%	90.63%	92.3%	87.62%	100%	100%	100%	98.98%	97.97%
Posip 076	93.31%	96.98%	98.32%	91.63%	92.97%	92.97%	92.97%	100%	98.98%
Vugava 115	92.3%	98.99%	96.32%	90.3%	90.96%	90.96%	90.96%	96.65%	100%
	Babic 015	Glavinusa 108	Marastina 058	Nincusa 108	Plavac mali 085	Plavac mali 128	Plavac mali 234	Posip 076	Vugava 115
**Coat protein (48V/49C)**									
Babic 015	100%	98.95%	100%	100%	98.95%	98.95%	N/A	100%	96.87%
Glavinusa 108	97.57%	100%	98.95%	98.95%	97.91%	97.91%	N/A	98.95%	97.91%
Marastina 058	96.88%	97.23%	100%	100%	98.95%	98.95%	N/A	100%	96.87%
Nincusa 108	96.88%	97.23%	100%	100%	98.95%	98.95%	N/A	100%	96.87%
Plavac mali 085	95.15%	94.8%	94.8%	94.8%	100%	100%	N/A	98.95%	95.83%
Plavac mali 128	95.15%	94.8%	94.8%	94.8%	100%	100%	N/A	98.95%	95.83%
Plavac mali 234 *	N/A	N/A	N/A	N/A	N/A	N/A	100%	98.95%	95.83%
Posip 076	95.15%	94.8%	94.8%	94.8%	99.3%	99.3%	99.3%	100%	96.87%
Vugava 115	96.88%	97.92%	96.53%	96.53%	94.11%	94.11%	94.11%	94.11%	100%
	Babic 015	Glavinusa 108	Marastina 058	Nincusa 108	Plavac mali 085	Plavac mali 128	Plavac mali 234	Posip 076	Vugava 115

*—Did not give positive result with primers 48V/49V.

**Table 3:** Comparison of the partial helicase and partial coat protein nucleotide sequences from the Croatian GRSPaV isolates with their most similar isolates in the GenBank database. For this comparison, 299 nts of the helicase gene and 289 nts of the coat protein gene were used.

Croatian Isolate	Helicase				Coat Protein			
	GenBank Isolate	Country	% Identity	Accession No.	GenBank Isolate	Country	% Identity	Accession No.
**Babic 015**	BS	Canada	98.66	AY881627	AV95 VVSY2	Tunisia Brazil	100	LT855242 KT008377
**Glavinusa 108**	NV_60U OV_104I OV_104G OV_104A	South Africa South Africa South Africa South Africa	99.33	MG050684 MG050602 MG050601 MG050600	NV_48E NV_48D NV_25D NV_25C LN-YEM-1	South Africa South Africa South Africa South Africa China	98.96	MG050300 MG050299 MG050262 MG050261 KF731972
**Marastina 058**	Pagadebit 2–5	South Africa	97.99	MG050684	NV_09E	South Africa	99.65	MG050227
**Nincusa 108**	SK704-C	Slovakia	98.32	KX274276	NV_09E	South Africa	99.65	MG050227
**Plavac mali 085**	GRSPaV-1	-	99.67	AF057136	NV_05C E78-RSP LN-XY1–3 AFH-2 PG	South Africa Spain China China Italy	99.65	MG050219 KJ466314 KJ634636 MK867356 HE591388 *
**Plavac mali 128**	Pagadebit 2–5	Italy	97.99	DQ278627	NV_09E	South Africa	98.62	MG050227
**Plavac mali 234**	34 clone 2	France	100	MG938304	N/A **	N/A **	N/A **	N/A **
**Posip 076**	Pagadebit 2–5	Italy	98.32	DQ278627	NUB1_BR AMCF clone 2 NV_38U NV_38B MB_93B	Brazil France South Africa South Africa South Africa	100	MK804766 MG938310 MG050435 MG050288 MG050192 *
**Vugava 115**	NV_60U OV_104I OV_104G OV_104A	South Africa South Africa South Africa South Africa	99%	MG050684 MG050602 MG050601 MG050600	NV_60B NV_25D NV_25C NV_06B LN-YEM-1	South Africa South Africa South Africa South Africa China	98.96	MG050326 MG050262 MG050261 MG050220 KF731972 *

*—Since more than 5 different isolates had the same identity, only five were selected. **—The grapevine accession Plavac mali 234 did not give a positive result with the primer pair 48V/49C targeting the coat protein region.

**Table 4:** Viruses and viroids identified in 22 grapevine accessions from grapevine virus collection by high-throughput sequencing. Accessions are indicated by cultivar name and corresponding number in the collection. The grey area represents viruses previously analyzed by ELISA during 2007/08. ELISA results are displayed in red in the bottom row.

Grapevine Accession ID	ArMV	GFLV	GFkV	GLRaV-1	GLRaV-2	GLRaV-3	GVA	GVB	GVE	GVF	GVG	GVT	GLRaV-4	GLRaV-4 strain 5	GLRaV-4 strain 6	GLRaV-4 strain 9	GRSPaV	GDefV	GRLDaV	GBV-1	GRVFV	GSyV-1	GYSVd-1	GYSVd-2	HSVd
**Babica 122**			**+**	**+** **+**		**+** **+**	**+** **+**			**+**							**+**		**+**		**+**		**+**		**+**
**Babic 055**			**+**		**+** **+**	**+** **+**	**+** **+**										**+**				**+**	**+**			**+**
**Babic 063**			**+** **+**			**+** **+**	**+** **+**	**+** **+**				**+**					**+**					**+**	**+**		**+**
**Dobricic 021**		**+** **+**				**+** **+**	**+**			**+**			**+**				**+**								**+**
**Glavinusa 102**				**+** **+**		**+** **+**	**+** **+**			**+**							**+**		**+**	**+**	**+**		**+**		**+**
**Marastina 016**			**+**				**+** **+**						**+**		**+**		**+**						**+**		**+**
**Marastina 037**	**+**	**+** **+**	**+** **+**	**+** **+**													**+**				**+**		**+**		**+**
**Mladenka 160**				**+** **+**		**+** **+**	**+** **+**			**+**	**+**						**+**								**+**
**Plavac mali 010**						**+** **+**	**+** **+**	**+** **+**					**+**	**+**			**+**						**+**	**+**	**+**
**Plavac mali 084**		**+** **+**		**+**		**+** **+**	**+**										**+**		**+**				**+**		**+**
**Plavac mali 113**		**+** **+**		**+** **+**			**+** **+**											**+**							**+**
**Plavac mali 219**		**+** **+**	**+** **+**	**+** **+**		**+** **+**	**+** **+**			**+**							**+**	**+**	**+**				**+**		**+**
**Plavac mali 235**		**+**		**+** **+**		**+** **+**	**+** **+**						**+**		**+**			**+**		**+**			**+**		**+**
**Plavac mali 275**		**+** **+**	**+** **+**	**+**			**+**										**+**						**+**		**+**
**Plavac mali 282**		**+** **+**		**+** **+**		**+** **+**	**+** **+**										**+**			**+**			**+**		**+**
**Plavac mali 313**						**+** **+**	**+** **+**													**+**			**+**		**+**
**Posip 056**						**+** **+**	**+**										**+**				**+**		**+**		
**Posip 084**			**+**			**+** **+**						**+**											**+**		**+**
**Posip 123**			**+**	**+** **+**													**+**						**+**		**+**
**Vlaska 150**			**+**			**+** **+**			**+**	**+**	**+**						**+**								**+**
**Vugava 036**		**+**	**+** **+**			**+** **+**											**+**								**+**
**Vugava 112**						**+** **+**	**+** **+**					**+**		**+**	**+**	**+**	**+**						**+**		**+**

ArMV, arabis mosaic virus; GFLV, grapevine fanleaf virus; GFkV, grapevine fleck virus; GLRaV-1, -2, -3, and -4, grapevine leafroll-associated viruses 1, 2, 3, and 4; GVA, B, E, F, G, and T, grapevine viruses A, B, D, E, F, G, and T; GRSPaV, grapevine rupestris stem pitting-associated virus; GDefV, grapevine deformation virus; GRLDaV, grapevine Roditis leaf discoloration-associated virus; GBV-1, grapevine badnavirus 1; GRVFV, grapevine rupestris vein-feathering virus; GSyV-1, grapevine Syrah virus-1; GYSVd-1, grapevine yellow speckle viroid 1; GYSVd-2, grapevine yellow speckle viroid 2; HSVd, hop stunt viroid.

**Table 5:** Results of screening for the presence of FD phytoplasma in different samples using LAMP during two growing seasons (2020 and 2021): −negative, +positive, N/A—not applicable/not tested, L—accession lost/died during previous season.

Grapevine Accession	FD-LAMP	
	2020	2021
Grk 003	−	N/A
Mladenka 148	−	N/A
Plavac mali 010	N/A	−
Plavac mali 011	N/A	−
Plavac mali 013	N/A	−
Plavac mali 105	N/A	−
Plavac mali 272	+	L
Plavac mali 313	+	+
Plavac mali 401	N/A	+
Posip 064	N/A	−
Vlaska 122	N/A	+
Vlaška 123	N/A	−
Vlaska 131	+	L
Vlaska 143	−	−
Vlaska 150	+	L

## Data Availability

All sequencing data of Croatian GRSPaV isolates obtained during the research were deposited in GenBank under accession numbers OM964879-87 for helicase and OM964888-95 for CP region.

## Supplementary Materials

The following are available online at https://www.mdpi.com/article/10.3390/plants11111485/s1, Figure S1: Detection of grapevine rupestris stem pitting-associated virus (GRSPaV) using Western blot (antibodies As7-276 and As2003) and RT-PCR (primer pairs RSP13/RSP14 and 48V/49C); Table S1: List of eight grapevine viruses determined by ELISA in grapevine accessions from grapevine virus collection; Table S2: Viruses and viroids identified in 22 grapevine accessions from grapevine virus collection by high-throughput sequencing.
