# Supplementary material for: Virus and Virus-like Pathogens in the Grapevine Virus Collection of Croatian Autochthonous Grapevine Cultivars
Source: Plants (Basel). 2022 May 31;11(11):1485. doi: 10.3390/plants11111485 (PMC9182833; doi:10.3390/plants11111485)
Supplement: Supplementary file 1 [file plants-11-01485-s001.zip › Supplementary Figure S1.pdf]

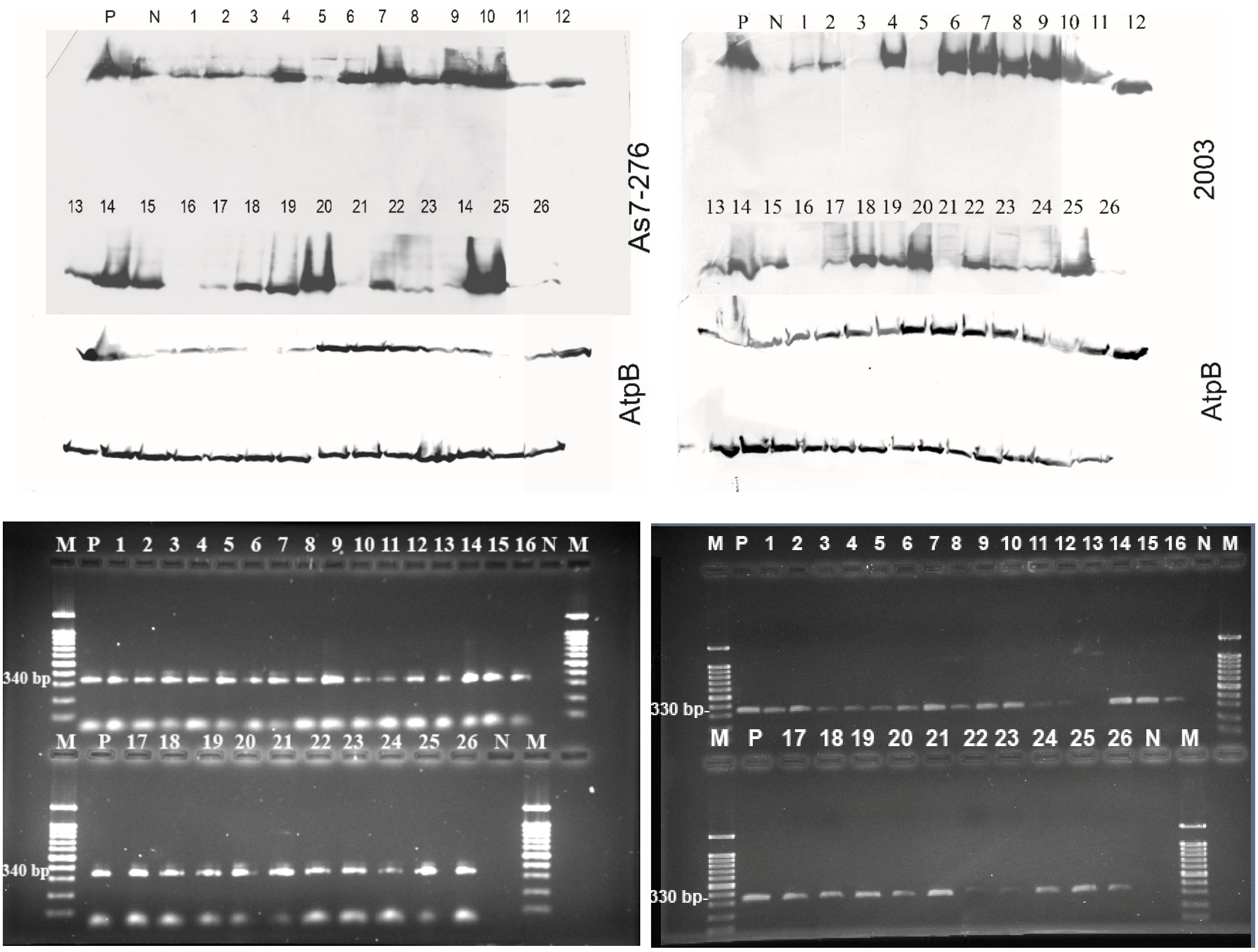

Supplementary Figure S1. Detection of grapevine rupestris stem pitting-associated virus (GRSPaV) using Western blot (antibodies As7-276—top left and As2003—top right) and RT-PCR (primer pairs RSP13/RSP14, expected product size ca 340 base pairs (bps) —bottom left, and 48V/49C, expected product size ca 330 bp—bottom right). P—positive control, N—negative control, AtpB—loading control, M—100 bp DNA marker (Intron Biotechnology, Gyeonggi-do, Korea), 1–26 different samples/grapevine accessions: 1—Babic 015, 2—Babic 056, 3—Babic 113, 4—Babic 076, 5—Marastina 034, 06—Marastina 058, 7—Marastina 101, 8—Pošip 076, 9—Vugava 060, 10—Vugava 115, 11—Plavac mali 013, 12—Plavac mali 085, 13—Plavac mali 234, 14—Plavac mali 286, 15—Plavac mail 111, 16—Plavac mali 128, 17—Plavac mali 159, 18—Plavac mali 181, 19—Plavac mali 219, 20—Babica 106, 21—Dobricic 101, 22—Glavinusa 110, 23—Ljutun 167, 24—Mladenka 154, 25—Nincusa 108, and 26—Vlaska 137. Only grapevine accessions 5, 16, and 21 reacted negative in Western blot with week signals in accessions 3 (As7–276) and 26 (both antibodies). According to RT-PCR, presence of virus was confirmed in all accessions with RSP13/RSP14 primers, while pair 48V/49C failed in detection only in grapevine accession 13.
